# Potential implications of Apolipoprotein E in early brain injury after experimental subarachnoid hemorrhage: Involvement in the modulation of blood-brain barrier integrity

**DOI:** 10.18632/oncotarget.10821

**Published:** 2016-07-24

**Authors:** Jinwei Pang, Yue Wu, Jianhua Peng, Ping Yang, Li Kuai, Xinghu Qin, Fang Cao, Xiaochuan Sun, Ligang Chen, Michael P. Vitek, Yong Jiang

**Affiliations:** ^1^ Department of Neurosurgery, The Affiliated Hospital of Southwest Medical University, Luzhou, China; ^2^ Departement of Neurosurgery, The First Affiliated Hospital of Chongqing Medical University, Chongqing, China; ^3^ Department of Vasculocardiology, The Affiliated Hospital of Southwest Medical University, Luzhou, China; ^4^ Department of Ophthalmology, The Affiliated Hospital of Southwest Medical University, Luzhou, China; ^5^ Department of Neurovascular Disease, The Affiliated Hospital of Zunyi Medical College, Zunyi, China; ^6^ Department of Medicine (Neurology), Duke University Medical Center, Durham, North Carolina, United States

**Keywords:** subarachnoid hemorrhage, Apolipoprotein E, early brain injury, blood-brain barrier, neuroinflamamation, Pathology Section

## Abstract

Apolipoprotein E (*Apoe*) genetic polymorphisms have been implicated in the long term outcome of subarachnoid haemorrhage (SAH), but little is known about the effect of *Apoe* on the early brain injury (EBI) after SAH. This study investigated the potential role of APOE in EBI post-SAH. Multiple techniques were used to determine the early BBB disruption in EBI post-SAH in a murine model using wild-type (WT) and *Apoe*^−/−^ (KO) mice. Progressive BBB disruption (Evans blue extravasation and T2 hyperintensity in magnetic resonance imaging) was observed before the peak of endogenous APOE expression elevation at 48h after SAH. Moreover, *Apoe*^−/−^ mice exhibited more severe BBB disruption charcteristics after SAH than WT mice, including higher levels of Evans blue and IgG extravasation, T2 hyperintensity in magnetic resonance imaging, tight junction proteins degradation and endothelial cells death. Mechanistically, we found that APOE restores the BBB integrity in the acute stage after SAH via the cyclophilin A (CypA)-NF-κB-proinflammatory cytokines-MMP-9 signalling pathway. Consequently, although early BBB disruption causes neurological dysfunctions after SAH, we capture a different aspect of the effects of APOE on EBI after SAH that previous studies had overlooked and open up the idea of BBB disruption as a target of APOE-based therapy for EBI amelioration research in the future.

## INTRODUCTION

Despite intense clinical efforts including surgical clipping and interventional embolization, subarachnoid haemorrhage (SAH) remains a devastating condition with case fatality rates of approximately 50% and one third of survivors requiring lifelong care [1]. Although the patients' neurological outcome is mainly determined by the severity of initial bleeding [2], secondary brain injuries after bleeding also contribute to the high mortality and disability [3, 4]. Considering no interventions for the prevention of SAH currently exist [5], pharmacotherapy has an accepted role in reducing the secondary brain injuries post-SAH.

Traditionally, cerebral vasospasm (CVS) induced delayed cerebral ischemia (DCI) was defined the primary cause of SAH induced poor outcome of patients [6]. However, the results of most pharmacologic interventions focused on the prevention and/or alleviation of CVS failed in the outcome improvement of SAH patients or just inconclusive [5]. Early brain injury (EBI) associated with SAH presents pathological brain changes including blood-brain barrier (BBB) disruption and others that arise within the first 72h following bleeding. These changes are now being recognized as the predominant determination of mortality and disability after SAH in humans [7]. Currently, however, therapeutic options adequately address this process are still limited [8].

Apolipoprotein E(APOE: protein, *Apoe: gene*) is the major apolipoprotein that is abundantly secreted by astrocytes in the central nervous system. APOE displays a strong BBB stabilization property [9], which may be beneficial for ameliorating EBI and stimulating subsequent improvements of neurological outcomes of SAH patients. Going further, the clinical translational potential of APOE-based research post-SAH lies in the fact that a series of APOE mimetic peptide have been demonstrated to be efficient neuroprotective agents [10, 11].

Therefore, we hypothesized that APOE was involved in EBI post-SAH through modification of the integrity of the BBB integrity. To approach this idea, we now describe behavioural, histological, molecular biological and ultrastructural data showing changes in BBB following SAH and the underlying molecular mechanisms that depend upon the presence or absence of APOE.

## RESULTS

### SAH severity and animal mortality

**Figure 1:**
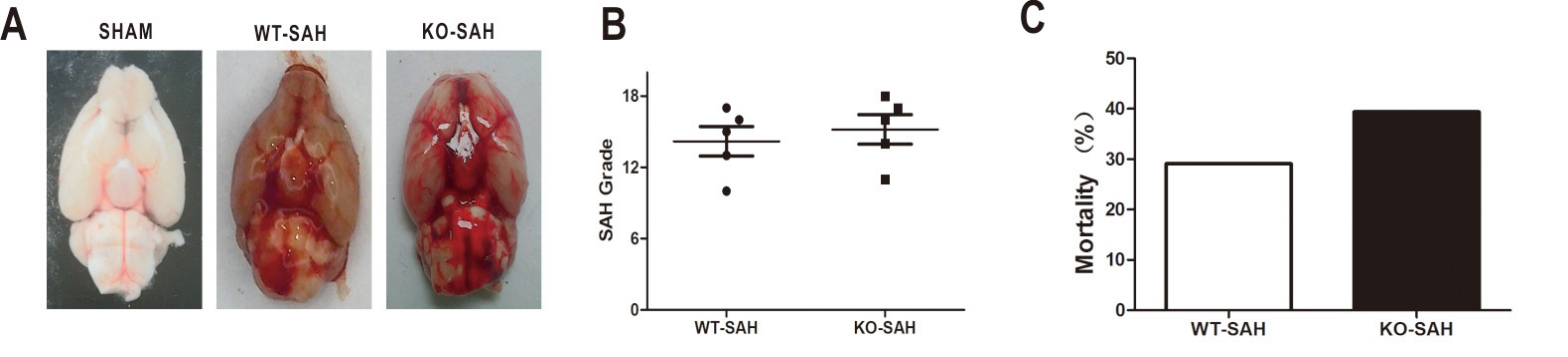
SAH grade and overall mortality Data are expressed as mean ± SEM. **A.**-**B.**, SAH grade in WT group and *Apoe* KO group were not significantly different. **C.** The overall mortality rate within 72h after SAH was 28.57% (34 of 119) in the WT group and 39.02% (16 of 41) in the KO group, with nonstatistical difference.

The SAH grade was determined by scoring as previously described [12] and did not reveal any significant difference between WT and Apoe-deficient mice. No animal died in the WT-SHAM, WT-SHAM+ saline and KO-SHAM groups. The overall mortality rate up to 72h after SAH was 28.57% (34 of 119) in the WT group and 39.02% (16 of 41) in the KO group. Although the mortality in the KO group was higher than the WT group, however, we failed to make a significant statistic difference of morality between these groups (Figure 1, *p* > 0.05).

### Brain edema after SAH and neurobehavioral dysfunctions after SAH

**Figure 2:**
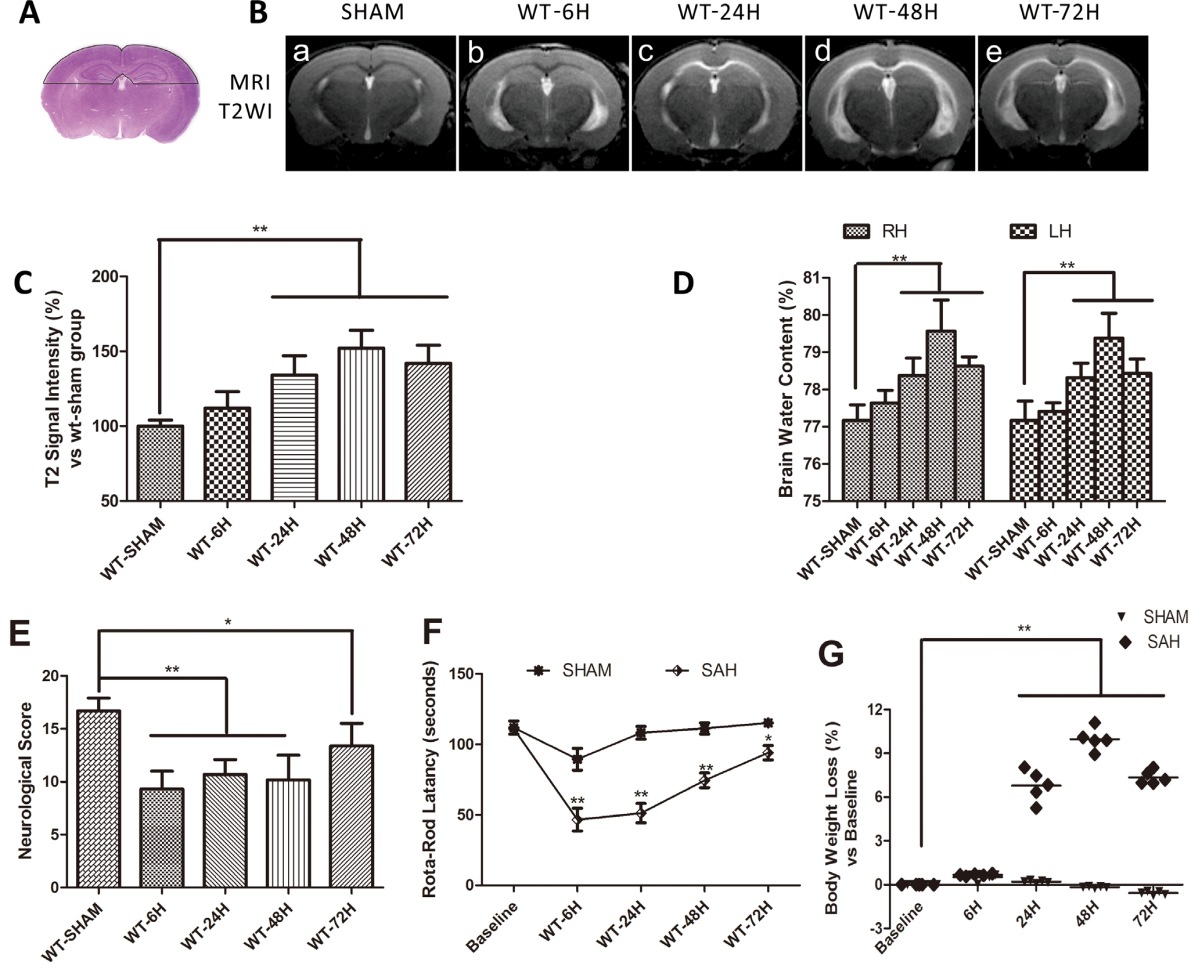
Vasogenic cerebral edema and neurobehavioral defects after SAH Data are expressed as mean ± SEM. **A.** The dotted area in represented the region of interest (ROI) contain the bilateral parietal lobe sensorimotor cortex and hippocampus sensitive to damage. The vasogenic cerebral edema was significantly increased at 24h and peaked at 48h (^**^P < 0.01, *n* = 5) **B.**-**C.**. Brain water content (BWC) resulted in the similar change (^**^P < 0.01, *n* = 5) **D.**, RH = right hemisphere, LH = left hemisphere). **E.**-**G.**The RR latency and Modified Garcia Score (MGS) decreased dramatically at 6h after SAH relative to their sham-operated counterparts. At later times, the rotorod (RR) latency and MGS performance gradually improved but continuously remained below the sham group levels. Meanwhile, compared with sham-operated mice, body weight loss progressively increased and peaked at 48h after SAH. (**P < 0.01,*P < 0.05 respectively, *n* = 5).

Brain edema is not only a predictor of EBI, but also a good parameter for assessment of BBB integrity. In the current study, T_2_ signal intensity values in the outlined region of interest (Figure 2A) gradually increased with time, peaking at 48h and then beginning to decline after SAH (Figure 2B, 2C) The brain water content (BWC) matched the T_2_ signal intensity value change with a peak at 48h after SAH and then began to decline (Figure 2D). (**p < 0.01,*p < 0.05 respectively, *n* = 5).

The RR latency and Modified Garcia Score (MGS) decreased dramatically at 6h after SAH relative to their sham-operated counterparts. At later times, the rotorod (RR) latency and MGS performance gradually improved but continuously remained below the sham group levels. Meanwhile, compared with sham-operated mice, body weight loss progressively increased and peaked at 48h after SAH (Figure 2E-2G). (**p < 0.01,*p < 0.05 respectively, *n* = 5)

### Endogenous APOE expression after SAH

**Figure 3:**
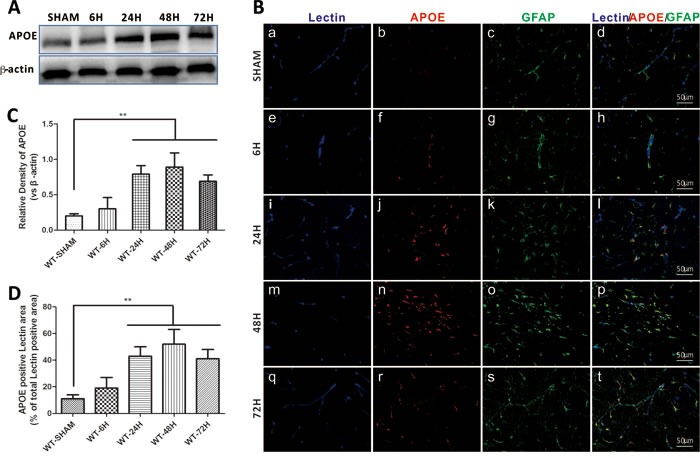
Endogenous APOE expression after SAH Data are expressed as mean ± SEM. In sham group, the endogenous APOE expression level was relative low in the astrocytes. After subarachnoid hemorrhage (SAH), APOE in the astrocytes were gradually elevated, up to 24h, the APOE expression was significantly up-regulated and peaked at 48h (^**^P < 0.01) **A.**-**C.**. The perivascular space APOE positive cells were significantly increased at 24h and 48h, provided an information indicating that APOE function in early brain injury (EBI) is associated with the BBB integrity modulation after SAH (^**^P < 0.01) **C.**, **D.**. (Scale bars = 50μm, Blue lectin positive area indicate microvessels; green GFAP positive area indicate astrocytes; red APOE positive area indicate APOE; *n* = 5 each group).

Compared to sham treated mice, the expression of APOE began to elevate at 6h after SAH induction. By 24h, APOE was significantly increased and peaked at 48h. (Figure 3A, 3C). The immunofluorescent staining of APOE also continuously increased and peaked at 48h. In addition, a significantly increased colocalization of glial fibrillary acidic protein (GFAP) positive APOE immunoreactive cells with lectin positive microvessels provided important information that APOE function may be associated with the BBB integrity after SAH. (Figure 3B, 3D). (**p < 0.01,*p < 0.05 respectively, *n* = 5).

### *Apoe* deficiency affects neurological dysfunctions after SAH

**Figure 4:**
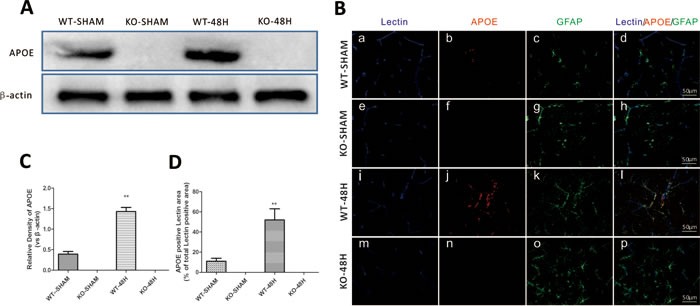
*Apoe* deletion affects mice neurological defects after SAH Data are expressed as mean ± SEM. The APOE protein expression was totally depleted in *Apoe*-deficient mice as shown by both western blot analysis **A.**, **C.** and immunofluorence staining **B.**, **D.**while the WT mice showed a significant APOE upregulation 48h after SAH (**P < 0.01). (Scale bars = 50μm, Blue lectin positive area indicate microvessels; green GFAP positive area indicate astrocytes; red APOE positive area indicate APOE; *n* = 5 each group). There was no significant difference in body weight loss (BWL), RR latency, MGS or brain edema between the sham groups (WT-SHAM *vs* KO-SHAM, @ means *p* > 0.05, *n* = 5). During the experimental lesion, *Apoe*-deficient mice displayed more BWL, shorter RR latency and less MGS compared with WT mice **E.**-**G.**. (WT-48H *vs* KO-48H, **p < 0.01,*p < 0.05 respectively, *n* = 5).

To further identify the involvement of APOE in EBI following SAH, we elucidated the neurological defects in *Apoe^−/−^* mice at 48h after SAH. Endogenous APOE expression was absent in the KO group mice (Figure 4A-4D). There was no significant difference in body weight loss (BWL), RR latency, MGS or brain edema between the sham groups (WT-SHAM vs KO-SHAM, @ means *p* > 0.05, *n* = 5). During the experimental lesion, *Apoe*-deficient mice displayed more BWL, shorter RR latency and less MGS compared with WT mice (Figure 4E-4G). (WT-48H vs KO-48H, **p < 0.01,*p < 0.05 respectively, *n* = 5). These data strongly support the hypothesis that APOE contributes to the process of EBI following a SAH insult to the brain.

### Apoe deficiency affects BBB integrity after SAH

**Figure 5:**
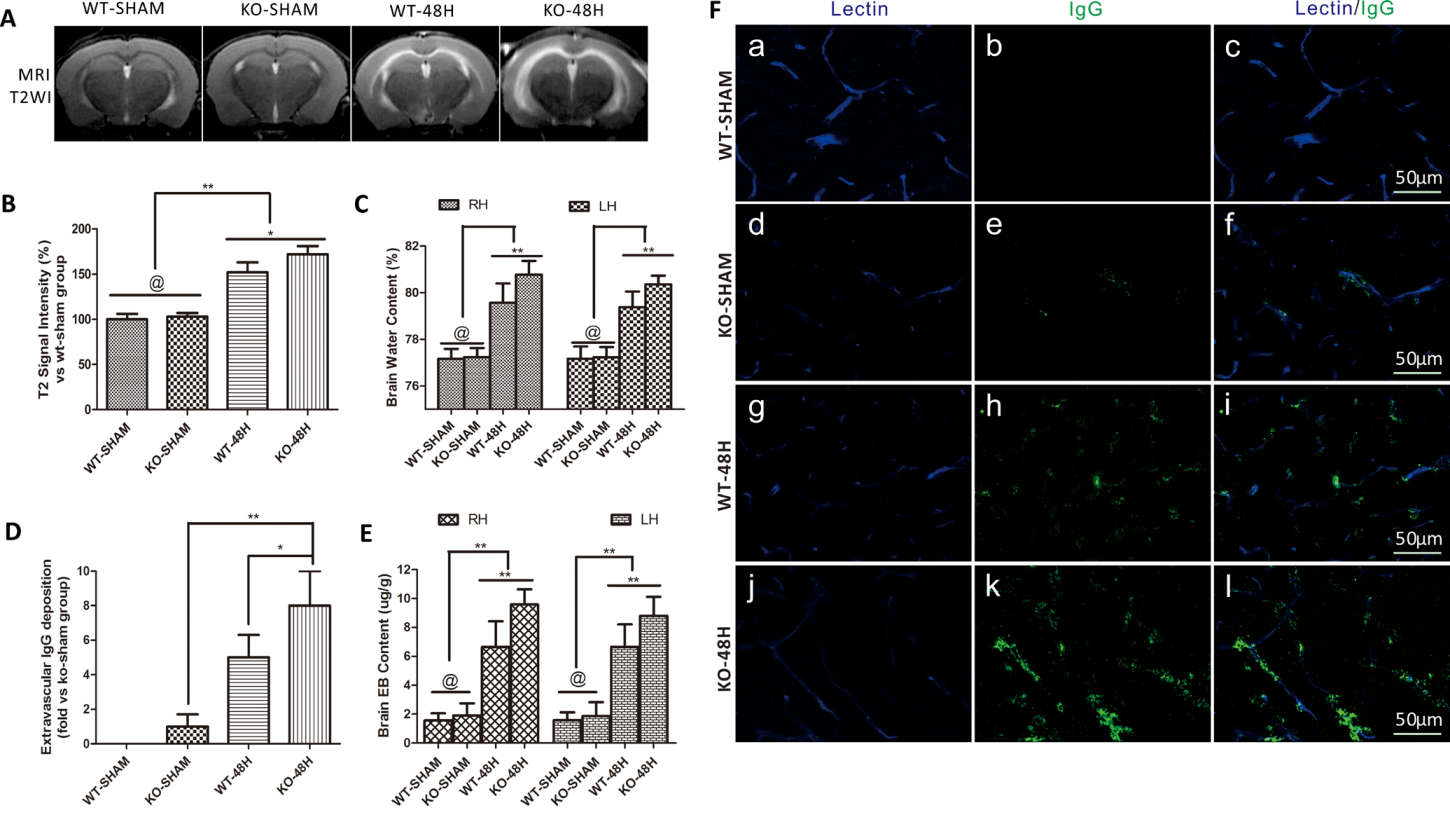
*Apoe* deficiency aggravates vasogenic brain edema and BBB permeability Data are expressed as mean ± SEM. **A.**-**C.** The Vasogenic cerebral edema and brain water content were not significantly deferent from each other in the sham groups (WT-SHAM *vs* KO-SHAM, @ represents that *p* > 0.05, *n* = 5). However, after SAH, T_2_-weight image (T2WI) MRI detected vasogenic cerebral edema in the prior dotted area and brain water content were dramatically deferent from each other with a higher edema level in the *Apoe*-deficient mice. The BBB permeability was not significantly different in the sham group detected by Evans blue (EB) extravasation **D.** and plasma immunoglobulin G immunofluorence staining **E.**-**F.**. However, both the brain EB content and extravascular IgG deposition were significantly elevated with higher levels far from the wild-type mice in *Apoe*-deficient mice (^*^P < 0.05, ^**^P < 0.01, *n* = 5 respectively, RH = right hemisphere, LH = left hemisphere, Blue lectin positive area indicate microvessels; green area indicate perivascular deposition of IgG; *n* = 5 each group).

A previous report showed that APOE controls the BBB integrity under physiological conditions [9]. To determine whether APOE controls the BBB integrity after SAH, we measured brain edema and BBB disruption in *Apoe^−/−^* mice at 48h after SAH. There was no significant difference of brain edema before SAH (WT-SHAM vs KO-SHAM). However, the T_2_-hyperintensity and BWC was dramatically higher in the *Apoe*-deficient mice after SAH (Figure 5A-5C, WT-48H vs KO-48H, @ means *p* > 0.05, **p < 0.01 and *p < 0.05 respectively, *n* = 5).

Evans blue (EB) has a high affinity with plasma albumin. After SAH, BBB integrity was damaged as evidenced by observation of albumin leakage and localization around the blood vessels [13]. In the current study, we found that EB extravasation was significantly increased at 48h after SAH while *Apoe*-deficient subjects exhibited a greater brain EB content as shown in Figure 5D (WT-48H *vs* KO-48H, **p < 0.01,*p < 0.05 respectively, *n* = 5).

Different from the EB extravasation, the IgG molecular weight is far larger than albumin and IgG extravasation represent a further disruption of the BBB [14]. IgG immunofluorescence staining was also significantly increased at 48h after SAH with greater levels in *Apoe*-deficient mice which suggest a more severe BBB disruption (Figure 5E-5F, **p < 0.01,*p < 0.05 respectively, *n* = 5).

### Apoe deficiency affects BBB structure after SAH

To make sure that APOE affects the BBB structure after SAH, we examined the expression of the major tight junction proteins (TJs), BBB transmission electron microscopy (TEM) scanning, and endothelial cells apoptosis status in the brain microvessles.

**Figure 6:**
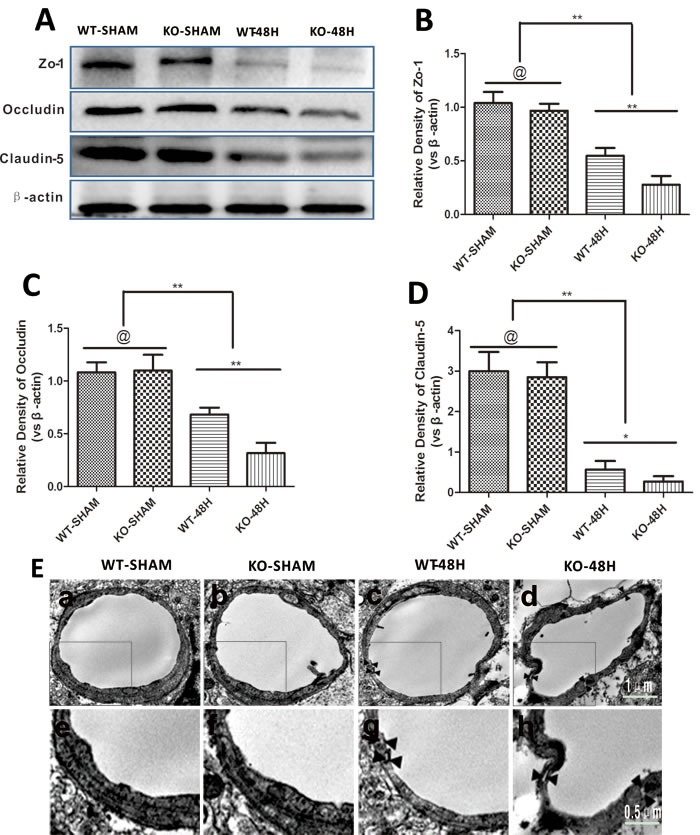
*Apoe* deficiency aggravates BBB structure damage Data are expressed as mean ± SEM. **A.**-**D.** The three major TJs were simultaneously decreased at 48h post-SAH. Furthermore, the decrease of TJs was more obvious in the KO-48H group (@ represents that *p* > 0.05, ^*^P < 0.05, ^**^P < 0.01 respectively, *n* = 5). **E.** TEM scanning showed TJs disruption and vacuolations between endothelial cells as pointed by the black arrows. In the *Apoe*-deficient mice group, more TJs disruption and bigger vacuolations between endothelial cells could be observed accompanied with microvessels lumen deformation (subjacent figures represent the dotted area in the corresponding ones above).

The three main TJs were not significantly different between the sham groups (WT-SHAM *vs* KO-SHAM, @ represents *p* > 0.05, *n* = 5). However, TJs were dramatically reduced 48 h after SAH and further affected by Apoe deficiency (WT-48H vs KO-48H, **p < 0.01,*p < 0.05, *n* = 5). Similarly, TEM observation showed more disruptions in TJs and vacuolations between endothelial cells (Figure 6).

**Figure 7:**
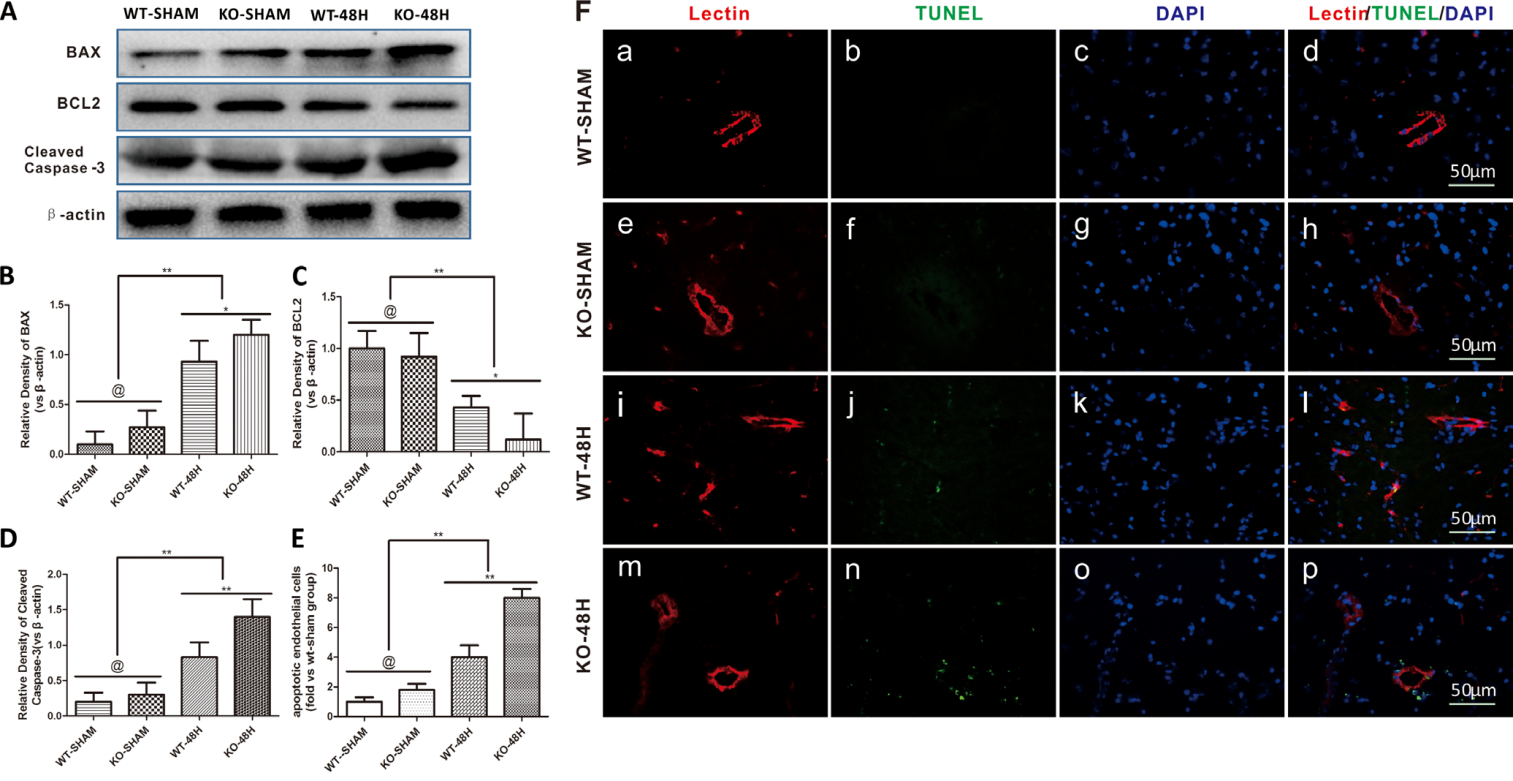
*Apoe* deficiency aggravates endothelial apoptosis Data are expressed as mean ± SEM. **A.**-**D.** In comparison with wild-type mice, the apoptotic related protein BAX, Cleaved Caspase-3 expressions were higher in the *Apoe*-deficient mice while the antiapoptosis protein BCL2 was significantly lower (@ represents that *p* > 0.05, *p < 0.05, **p < 0.01 respectively, *n* = 5). **E.**-**F.**. *Apoe*-deficient mice displayed more endothelial cells death detected by Lectin and TUNEL double stainning (Red lectin positive area indicate microvessels; blue area indicate TUNEL and blue DAPI area indicate nucleus; @ represents that *p* > 0.05, ^*^p < 0.05, ^**^p < 0.01 respectively, *n* = 5).

Endothelial cells, another main component of the BBB, was also further injured in *Apoe*-deficient mice at 48 h after SAH. Apoptosis-related proteins including BAX and Cleaved Caspase-3 were significantly upregulated, while the anti-apoptotic protein BCL2 was decreased after SAH. This phenomenon was more pronounced in *Apoe*-deficient mice, which resulted in increased apoptosis of endothelial cells as shown in Figure 7 (@ represents *p* > 0.05, **p < 0.01,*p < 0.05, *n* = 5).

### *Apoe* deficiency causes enhanced MMP-9 upregulation after SAH

**Figure 8:**
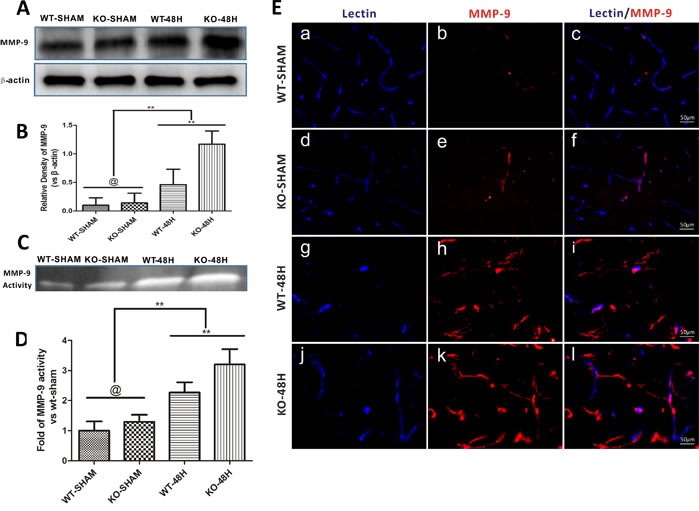
*Apoe* deficiency aggravates MMP-9 upregulation after SAH Data are expressed as mean ± SEM. **A.**-**B.**The protein expression analysed using western blotting demonstrated a greater levels of MMP-9 after SAH in *Apoe*-deficient mice when compared with WT ones. **C.**-**D.** The MMP-9 enzymatic activity was also higher in *Apoe*-deficient mice at 48h after SAH. **E.**The immunofluoresence stain showed that the perivascular MMP-9 location was abundantly increased after SAH, perivascular MMP-9 positive area was more obvious in the *Apoe*-deficient mice. (Blue lectin positive area indicate microvessels; red area indicate perivascular location of MMP-9; @ represents that *p* > 0.05, ^**^p < 0.01 respectively, *n* = 5).

MMP-9 is well studied for the role that it plays in the degradation of BBB TJs and endothelial cells apoptosis [15, 16]. Prior to SAH, both the expression and activity of MMP-9 was not obviously different between the two sham groups (WT-SHAM *vs* KO-SHAM, @ represents *p* > 0.05, *n* = 5). However, after SAH, both the expression and activity of MMP-9 was upregulated, with more severe changes in *Apoe^−/−^* mice (WT-48H *vs* KO-48H, **p < 0.01,*p < 0.05, *n* = 5) (Figure 8A-8D). Moreover, the perivascular MMP-9 elevation was also significantly higher in the *Apoe*-deficient mice (Figure 8E).

### *Apoe*-deficient mice displayed enhanced inflammatory cytokines production after SAH

**Figure 9:**
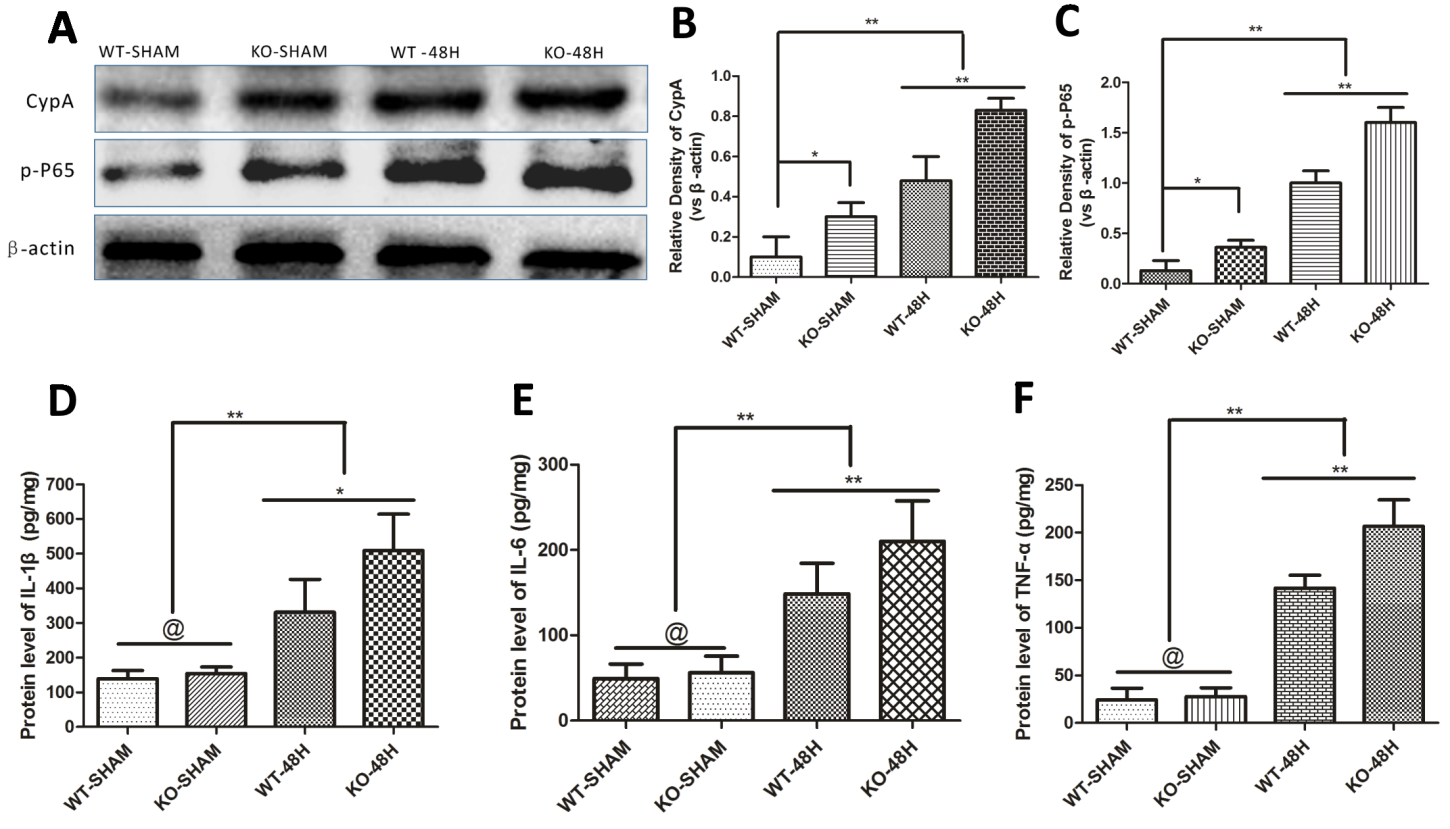
*Apoe* deficiency aggravates the inflammatory response Data are expressed as mean ± SEM. Although the main mediators of MMP-9 like CypA and phosphorylated nuclear factor kappa B subunit p65 (p-P65) were slighty different in the sham group, the discrepancy has greater statistical significance at 48h after SAH (^*^P < 0.05, ^**^P < 0.01 respectively, *n* = 5) **A.**-**C.**. The enzyme-linked immuno sorbent assay (ELISA) measurement of TNF-α, IL-6 and IL-1β, another three inflammatory mediators of MMP-9 downstream the CypA- NF-κB related pathway, showed no significant difference of these inflammatory cytokines. Nevertheless, the discrepancies of these cytokines have greater levels at 48h after SAH in *Apoe*-deficient mice compared with WT mice (@ represents that *p* > 0.05, ^**^P < 0.01 respectively, *n* = 5) **D.**-**F.**.

The inflammatory response is considered one of the main causes of MMP-9 upregulation after SAH [17, 18]. In the current study, CypA, p-p65 were slightly higher in the *Apoe*-deficient mice whereas IL-1β, IL-6 and TNF-α levels were not significantly different in the two control groups before SAH (WT-SHAM vs KO-SHAM, *p < 0.05, @ represent *p* > 0.05, *n* = 5). However, both CypA, p-p65, IL-1β, IL-6 and TNF-α were dramatically up-regulated 48h after SAH with larger increases of these proteins in *Apoe*-deficient mice (WT-48H vs KO-48H, **p < 0.01,*p < 0.05 respectively, *n* = 5). (Figure 9)

## DISCUSSION

In this study, we defined a therapeutic target for EBI inhibition to restrict neurological dysfunctions in experimental SAH. This approach is based on our results showing that: (1) endogenous APOE is significantly elevated at early times after SAH, (2) APOE elevation is associated with EBI inhibition through a mechanism that includes BBB-dependent changes.

Recently, emerging data support the multiple neuroprotective properties of APOE with combined anti-inflammatary [19], anti-apoptosis [20] and anti-oxidant [21] effects, all of which may be beneficial for EBI inhibition. However, evidence also exists that support a lack of effect of APOE on neuronal cells following injury [22]. At present, no direct research focused on the involvement of APOE in the pathogenesis of EBI post-SAH has been reported. Additionally, APOE expression levels change after brain injury, although different reports suggest APOE levels can be increased [23] or decreased [24]. Nevertheless, APOE changes after SAH has thus far not been described nor whether this change, if it occurs, is beneficial, pernicious or neutral has been addressed.

Strikingly, we identified that the expression of APOE began to rise as early as 6h after SAH induction. By 24h post-injury, APOE was significantly increased and then peaked at 48h, followed by a gradual decline toward the baseline at 72h post-injury in the cortex. In our observation, this endogenous APOE protein level change was tightly associated with the mice neurological dysfunctions after SAH. The mice neurobehavioral functions were significantly destroyed than the sham-operated mice before the peak of APOE expression. Once the APOE reached a high level, these neurobehavioral dysfunctions recovered quickly toward the baseline. Moreover, there was no significant difference in both body weight loss (BWL), RR latency, MGS and brain edema between the sham-operated groups. However, during the experimental lesion, *Apoe*-deficient mice displayed more BWL, shorter RR latency and less MGS compared with WT mice after SAH. As a whole, these data strongly indicate that APOE is of significant benefit during the period of EBI following SAH in helping to restore normal neurological functions.

BBB disruption is an early feature of EBI. It was described as a hallmark that significant contribute to the poor outcomes of SAH patients [25]. Links between APOE and BBB integrity have been previously reported. For instance, Bell et al. [9] demonstrated that APOE controls the cerebrovascular integrity via cyclophilin A (CypA) in the aging transgenic mouse brains under physiological conditions. Zhang et al. [26] also confirmed that *Apoe*-deficiency promotes BBB disruption in experimental autoimmune encephalomyelitis *via* alteration of MMP-9. However, the BBB disruption observed in the above reports are more of a chronic inflammatory response, whereas SAH induced BBB disruption is an acute response and controlled by complex mechanisms (e.g. intracranial hypertension, global brain ischemic, neuroinflammation).

From this perspective, we started to question whether the action of APOE on EBI is through modulation of BBB integrity after SAH. To explore this possibility, we used multiple techniques to reveal the functional and structural changes of BBB integrity after SAH. We found progressive increase of both brain EB content, immunoglobulin G extravasation and T2 hyperintensity in magnetic resonance imaging before the peak of endogenous APOE expression at 48h after SAH in the WT mice. *Apoe^−/−^* mice exhibited more severe degrees of these functional BBB disruption charcteristics. In addition, the protein levels the major tight junction proteins (TJs) were dramatically reduced 48h after SAH and further affected by *Apoe* deficiency. Similarly, TEM observation showed more disruptions in TJs and vacuolations between endothelial cells. Simultaneously, Apoptosis-related proteins including BAX and Cleaved Caspase-3 were significantly upregulated, while the anti-apoptotic protein BCL2 was decreased after SAH. This phenomenon was more pronounced in *Apoe*-deficient mice, which resulted in increased apoptosis of endothelial cell. Taken together, these data demonstrated a significant crucial role for APOE in BBB stabilization post-SAH.

Although we found that APOE plays an important role in EBI post-SAH through the modulation of BBB integrity, the mechanism by which APOE could influence the response to BBB disruption after SAH have not being directly explored before. There are several possible interpretations of these results. Firstly, inflammation is one of the main mechanisms associated with BBB damage [17]. Bell et al. [9] demonstrated that APOE controls BBB integrity *via* suppressing the inflammatory CypA-NF-κB-MMP-9 pathway in the physiological aging brain. However, there is also support for increased expression of CypA that may play a protective role against BBB disruption after traumatic brain injury (TBI) [27]. Whether APOE related CypA inhibition is beneficial for the early BBB stabilization after SAH remains unclear. Thus, in search of the molecules that contribute to APOE-mediated BBB integrity in EBI post-SAH, we focused on the proinflammatory cytokine CypA.

Specifically, protein levels of CypA, its downstream target phosphorylated NF-κB subunit P65 (p-P65) and MMP-9 are very low in the sham-operated groups, whereas significant increases were observed after SAH. The activation of CypA was followed by the NF-κB increase and release of abundant proinflammatory cytokines (including IL-6, TNF-α and IL-1β) and MMP-9 upregulation. *Apoe*-deficient mice exhibited more exaggerated levels of CypA, p-P65 and MMP-9 after SAH when compared with WT mice. The increase of NF-κB is involved in the transcriptional activation of MMP-9 in cerebral vessels [28]. NF-κB could also transcriptionally trigger the secretion of proinflammatory cytokines like IL-6, TNF-α and IL-1β [29, 30], which have been previously reported to activate MMP-9 [17].

In the current study, the very low levels of CypA was related to a normal BBB integrity due to less p-P65 and proinflammatory cytokines including IL-6, TNF-α and IL-1β, whereas the increased CypA was associated with a significant disruption of BBB integrity due to increased p-P65 and proinflammatory cytokines release in the WT mice. In the sham operated *Apoe*-deficient mice, CypA was slightly higher than the WT mice and the TJs were slightly fewer than the WT mice. However, the BBB permeability was not significantly different from the WT mice. These non-statistic difference of CypA related BBB integrity may be due to the very young age of mice since previous study reported that Apoe deficiency associated BBB disruption in Alzheimer's disease, without any other acute injury effects, was *age*-dependent [31]. Therefore, taken these data together, we favor the hypothesis that APOE is indispensable for proper CypA levels in maintaining normal BBB integrity through the direct CypA-NF-κB-MMP-9 pathway under physiological conditions. However, aberrant upregulation of CypA impairs BBB integrity after SAH due to the significant increase of proinflammatory cytokine release while APOE could suppress this detrimental effect through the indirect CypA-NF-κB-proinflammatory cytokines-MMP-9 pathway.

In addition, the direct transcriptional modulation of APOE on inflammatory cytokines maybe also an explanation. Study revealed that APOE could enter into the nuclear and exist a direct transcriptional effect [32]. In this paper, Theendakara V et al. declared that the transcriptional manner of APOE was tightly associated with the expression levels of inflammatory cytokines including NF-κB and IL-6. We also observed a continuous increase of neucleus APOE protein level after SAH (data not shown). However, whether the presently reported inflammatory suppression properties of APOE were simply through the above mentioned mechanisms or combined with transcriptional effect are not tested this time. Later studies should take in technics that analysis the transcriptional effect of APOE on EBI post-SAH.

Another possible mechanism involves endothelial cell apoptosis. Inflammation often results in endothelial cell apoptosis [33]. Previously, CypA was demonstrated to be involved in endothelial cell dysfunctions [34]. In the current study, endothelial cell apoptosis was significantly increased after SAH. Paralleled with the increase of CypA, apoptosis-associated proteins like BAX and Cleaved Caspase-3 were also significantly upregulated, while the anti-apoptotic protein BCL2 was significantly decreased. These changes were more serious in the absence of APOE, which indicated that the reduction of CypA by APOE was also involved in endothelial cell apoptosis after SAH.

It should be noted that endothelial cell apoptosis is not always induced by inflammation. There are two independent apoptosis pathways including a mitochondrial-dependent and a non-mitochondrial-dependent pathways [35, 36]. Whether the suppression of apoptosis by APOE is the effect of inhibiting inflammation through a CypA-related pathway or through the direct suppression of apoptotic pathways is not fully understood this time. The detailed mechanisms remain to be further investigated.

Taken together, the present study investigated the neuroprotective effects of APOE in EBI following SAH and provided potential explanations for these observations. The next station is to investigate whether the APOE-based therapies, like APOE-peptide mimetics, have therapeutic values in attenuating EBI after SAH. However, there are still several limitations need to be mentioned.

Firstly, mice underwent the endovascular perforation model under anesthesia and experienced bleeding unconsciously, painlessly, and without fear. This situation is significantly different from that of clinical patients who experience bleeding while awake. To study the brain injuries after SAH further, SAH models in which animals are conscious are needed. Secondly, although the statement is still controversial, it was reported that *Apoe* polymorphisms were associated with the outcome of SAH patients, different alleles had different functions [37, 38]. However, the effects of APOE on EBI post-SAH were tested only in WT and *Apoe^−/−^* mice. Transgenic mouse models in which the human APOE alleles have been “knocked in” should be used in future studies. Thirdly, in order to reduce the individual difference factors, all mice used were 8-10 weeks-old young adult male C57BL/6J mice and condition matched *Apoe^−/−^*mice. The neurological functions and BBB integrity were not significantly different before the SAH, whereas the differences were significant after SAH injury. Whether there remain some other mechanisms involved in this process, or subject gender and age have effects on the protection of APOE in SAH was unclear.

In summary, this study is the first to our knowledge showing the significance of APOE in attenuating EBI after SAH through a BBB modulation-dependent manner that previous studies had overlooked and open up the idea of BBB disruption as a target of APOE-based therapy for EBI amelioration research. However, as this is an experiment using animal models, more preclinical studies are needed for a variety of variables that have yet to be investigated before our results can be extrapolated to a clinical trial in humans.

## MATERIALS AND METHODS

### Animals

Experimental animals were housed and cared for in the Laboratory Animal Resource Center (LARC) at Chongqing Medical University. All procedures were approved by the Institutional Animal Care and Use Committee (IACUC) at the Chongqing Medical University. Adult male wild-type C57BL/6J mice (WT; 9.1±0.6 weeks; 18.2±0.4g) or condition matched adult male *Apoe^−/−^* on a C57BL/6 background (KO; 8.9±0.2 weeks; 18.1±0.6g) were used.

### Experimental groups

This study included the following 2 parts:

#### Part1

wild-type (WT) mice were randomly assigned to each of the experimental groups to determine the impact of endogenous APOE expression on BBB disruption and outcomes at 6h, 24h, 48h and 72h after SAH (*n* = 15).

#### Part2

After demonstration of the peak of APOE expression 48 h post-SAH, we investigated the role of APOE in BBB restoration by comparing BBB disruption between WT and *Apoe^−/−^* mice 48 h after SAH using the following 4 groups: WT-SHAM, KO-SHAM, WT-48H and KO-48H (*n* = 25).

### Induction of SAH

To induce SAH in mice, endovascular perforation was employed to puncture the bifurcation of the right middle cerebral artery and anterior artery as previously reported [39]. Briefly, mice were anesthetized with 2% pentobarbital (50mg/kg) intraperitoneal injection. A 5-0 monofilament nylon suture (Ethicon, Somerville, NJ, USA) at the length of 10mm was pushed from the right External Carotid Artery (ECA) into right internal carotid artery (ICA) to perforate the bifurcation of the middle and anterior cerebral artery. In the sham group, all operational procedures were repeated except that the filament did not puncture the microvessle so that vessel rupture was avoided. Dead mice were replaced with condition matched littermates immediately. The body temperature was maintained at 37.5±0.5 ­during the operation.

### SAH severity and mortality analysis

All surviving mice after the surgery reveived a dissection and SAH grade alaysis. The SAH grade was determined by scoring as previously described [12]. Briefly, High-resolution pictures of the base of the circle of Willis and basilar arteries were taken. The basal cistern was divided into six segments and each segment was allotted a grade from 0 to 3 depending on the amount of blood clot. The total score of the six segment was defined as the severity of mice SAH grade.

Mortality was calculated as the dead mice percentage in the total number of mice after SAH in each group. Only the mice that score higher than 8 and the dead ones due to server bleeding were analyzed.

### Neurobehavioral dysfunction measurements

To investigate the influence of *Apoe* deficiency on neurobehavioral outcomes after SAH, scoring using a modified Garcia Scale (MGS) [40], body weight loss (BWL) [41] and Rota-Rod Latency (RR) [42] were carried out as previously reported. Briefly, for MGS analysis, six tests including spontaneous activity, spontaneous movement of four limbs, forepaw outstretching, climbing, body proprioception and response to whisker stimulation (318 points). The mean of neurologic score for grading was evaluated by two blinded observers. For RR Latency measurement, an automated rota-rod (ZB-200 Rota-Rod Treadmill; Taimeng Software Co. LTD, Chengdu, China) was used. The baseline of Rota-Rod Latency of each mouse was examined immediately before SAH induction with an accelerating speed (started from 0 rpm, accelerated by 3rpm every 10 seconds until the rotating speed reached 30 rpm) for three times and repeated after SAH induction. The Rota-Rod Latency is defined as the average time length of all three trials.

### Magnetic resonance imaging (MRI)

MRI with T_2_-weighted image analysis (T2WI) is a sensitive and reliable detection of vasogenic cerebral edema. Moreover, the main advantage of T2WI over histological methods is the noninvasive nature of the scanning permits the longitudinal monitoring of the evolution of BBB breakdown and the development of vasogenic edema over time in the same experimental subjects. For the continuous monitoring of BBB disruption and subsequent vasogenic cerebral edema, T_2_-weighted image (T2WI) MRI was performed after neurological evaluation as previously reported [10]. Briefly, MRI scans were performed using a Bruker 7.0T system (BrukerBiospin, Billerica, MA). Pre- and post-SAH mice were anesthetized with isoflurane. T2WI were acquired using RARE (repetition time = 4000, echo time = 45, RARE factor 8, 0.5mm, field of view 2.5 cm, 256×256). A region of interest (ROI) was defined to contain the bilateral parietal lobe sensorimotor cortex and the hippocampus, which are both sensitive to damage.

### Brain water content (BWC) calculations

After neurological evaluation and MRI scanning, BWC was measured as previously reported [43]. Briefly, after the animals were sacrificed with over dose of pentobarbital sodium, the brain samples were harvested and quickly separated into the left and right hemispheres. The brain specimens were immediately weighed to obtain the wet weight and then dried at 100°C for 72h before determining the dry weight. BWC was calculated according to the wet/dry method where BWC = (wet weight - dry weight)/wet weight×100 %.

### Evaluation of BBB permeability

After neurological evaluation, the BBB permeability was evaluated by extravasation of Evans blue dye (EB) following intravenous injection and plasma-derived IgG immunohistochemistry staining as previously reported and individually modified [28, 44]. Briefly, for EB extravasation analysis, 2% EB (4ml/kg, Sigma) was injected intravenously and kept the EB circulated for 3 hours. The animals were intracardially perfused with heparinised 0.9% saline. The brains were harvested and quickly separated into left and right hemispheres and weighted. EB dye was extracted from brain tissues using 50 % trichloroacetic acid and the optical density of supernatants was measured at 620 nm using a multi-mode microplate reader (Molecular Devices, USA). The brain content of EB was expressed as the content of EB dye quantity in the brain quantity (ug/g).

### Transmission electron microscopy (TEM)

Transmission electron microscopy (TEM) was carried out to visualize the BBB structural integrity as previously described [45]. Briefly, mice brain samples contain the bilateral parietal lobe sensorimotor cortex and the hippocampus were harvested under deep anesthesia and immediately sliced into 1mm^3^ slabs, post-fixed in glutaraldehyde overnight at 4°C. Subsequently, the tissues were fixed in 2% osmium, dehydrated in graded ethanol and flat-embedded in epon 812 (EMS Co., Ltd., Washington, USA). Thereafter, ultra-thin sections (50 nm) were made and double stained with uranyl acetate and lead citrate. Sections were then visualized under a Hitachi 7100 transmission electron microscope (Olympus, Tokyo, Japan).

### Western blotting analyses

Brain cortex from the right hemisphere of mice was extracted and Western blotted as previously described [10]. Primary antibodies included mouse monoclonal antibody against APOE (ab1907, Abcam), rabbit polyclonal antibody against MMP-9 (ab38898, Abcam), rabbit polyclonal anti-Zo-1 (ab59720, Abcam), rabbit polyclonal anti-claudin-5 (ab15106, Abcam), rabbit polyclonal anti-occludin (ab31721, Abcam), rabbit monoclonal anti-phospho-NF-kappaB-p65 (p-p65) (3033S, Cell Signaling Technology), rabbit polyclonal anti-BAX (50599-2-Ig, Proteintech), rabbit polyclonal anti-BCL2 (12789-1-AP, Proteintech), rabbit polyclonal anti-Cleaved Caspase-3 (#9664, Cell Signaling Technology) and mouse monoclonal anti-beta-actin (#SC-47778, Santa Cruz) were used on immunoblots.

### Gelatin zymography of MMP-9

The enzymic activity of MMP-9 was detected using a gelatin zymography Kit according to the manufacturer's protocol (GMS30071.1, Genmed Scientifics Inc. U.S.A).

### Enzyme-linked immunosorbent assay (ELISA)

Quantification of the protein levels of TNF-α, IL-1β and IL-6 were performed by ELISA measurement according to the manufacturer instructions of the ELISA kits (Boster, Wuhan, China). Briefly, homogenates of the right hemisphere of mice were prepared for detection according to the manufacturer's instructions of the ELISA kits. The protein content of each homogenate sample was detected by a BCA kit (Beyotime, Shanghai, China) and the content of each inflammatory cytokine was detected by the ELISA kits and normalized to protein levels.

### Immunofluorescence staining

APOE, MMP-9 and mouse IgG immunofluorescence staining were carried as previously described [46]. Briefly, mice were perfused with ice-cold phosphate buffered saline (PBS, pH = 7.4) followed by 4% paraformaldehyde (PFA) under deep anesthesia. The whole brains were harvested and coronary frozen sections (10μm) were made. Primary antibodies used included biotinylated lycopersicon esculentum (Tomato) Lectin (B-1175, Vector Laboratories;), mouse monoclonal antibody against APOE (ab1907, Abcam), rabbit polyclonal antibody against GFAP (16825-1-AP, Proteintech), mouse monoclonal antibody against MMP-9 (sc-13520, Santa Cruz) and goat polyclonal antibody against mouse IgG (115-005-003, Jackson). Second antibody used including AMCA Streptavidin (SA-5008, Vector Laboratories), DyLight 488 AffiniPure Goat Anti-Rabbit IgG (A23220, Abbkine), DyLight 594 AffiniPure Goat Anti-Mouse IgG (A23410, Abbkine) and DyLight 488 AffiniPure Rabbit Anti- Goat IgG (A23230, Abbkine).

### Immunofluorescence staining results analysis

Image-pro plus (IPP) 6.0 software was used for immunofluorescence staining results analysis. Three nonadjacent coronary sections in each brain sample with a minimum of 100 μm from each other were used and five randomly selected visual fields per section were analyzed. The Lectin-positive and APOE-positive area means the area of Lectin and APOE totally measured using IPP. APOE-positive Lectin area was defined while the area represents the expression of perimicrovascular APOE, it was also measured by area. Meanwhile, the extravasation of plasma IgG was measured by average accumulative fluorescence intensity calculation.

### Statistical analysis

Quantitative data was expressed as mean ± SEM and compared with repeated measures of one-way analysis of variance (one-way ANOVA). Bonferroni's post-hoc method was applied for comparison among groups. The mortality analysis was measured by Pearson Chi-Squared test. All statistical values were calculated using SPSS 19.0 software. (SPSS, Inc. Chicago, USA). A value of *p* < 0.05 was considered to be statistically significant.
